# Curcumin inhibits liver metastasis of gastric cancer through reducing circulating tumor cells

**DOI:** 10.18632/aging.101848

**Published:** 2019-03-07

**Authors:** Xixi Gu, Qiqi Zhang, Wei Zhang, Liang Zhu

**Affiliations:** 1 Department of Integrative Medicine, Zhongshan Hospital, University of Fudan, Shanghai 200032, China; 2 Department of Interventional Therapy, Zhongshan Hospital, University of Fudan, Shanghai 200032, China

**Keywords:** Curcumin circulating tumor cells (CTCs), primary gastric cancer (PGC), CXCR4, stromal cell -derived factor-1 (SDF-1)

## Abstract

Primary gastric cancer (PGC) is the fourth most common malignant human cancer and the second leading cause of death worldwide. The majority of the subjects of PGC is diagnosed at a late stage, resulting in poor prognosis and therapeutic outcome, largely attributable to dissemination of tumor cells into circulation as circulating tumor cells (CTCs) and their formation of distal tumor. Curcumin is an active ingredient from the rhizome of the plant Curcuma longa. Here, we assessed whether treatment with Curcumin may reduce the incidence of metastatic tumor formation in liver in mice carrying PGC. We found that Curcumin treatment significantly reduced the presence of CTCs and formation of liver tumor. Mechanistically, Curcumin reduced CXCR4 expression in PGCs in vitro and in vivo, and thus likely inhibited metastasis of PGC through suppression of stromal cell -derived factor-1/CXCR4 signaling. Thus, our study suggests that Curcumin may inhibit liver metastasis of PGC through reducing CTCs.

## Introduction

Gastric cancer (GC) is the fourth most common malignant human cancer and the second leading cause of death worldwide [1]. The majority of the subjects of GC is diagnosed at a late stage, resulting in poor prognosis and therapeutic outcome, largely attributable to dissemination of tumor cells into circulation as circulating tumor cells (CTCs) and their formation of distal tumor, especially in liver [2–4]. Besides presence in circulation, CTCs are identified with a number of surface markers, among which CXCR4, the receptor for stromal cell -derived factor-1 (SDF-1), is the best characterized one in different types of cancer [5–7]. In our previous report, we have studied the CTCs in hepatocellular carcinoma (HCC) and shown that CD90+CXCR4+ HCC cells may be CTCs and selective elimination of these cells may substantially improve the current HCC therapy by reducing cancer metastasis [8]. In the current study, we focused on the study of CTCs in GC.

Curcumin is an active ingredient from the rhizome of the plant Curcuma longa [9]. Interestingly, accumulating evidence suggest an anti-cancer function of Curcumin, and this potential effect of Curcumin has been suggested to work through suppression of growth of cancer cells by inhibition of multiple cell signaling pathways that regulate cell replication (e.g. through CCND1 [10], c-myc [11–14]), that regulate cell survival (e.g. through Bcl-2, Bcl-xL, cFLIP, caspase-8, 3, 9) [15–18], that regulate tumor suppressors (e.g. through p53, p21) [19–23], and that regulate protein kinase pathway (e.g. through JNK, Akt, and AMPK) [24–26]. However, whether Curcumin may affect the CTCs in GC is unknown.

In the current study, we assessed whether treatment with Curcumin may reduce the incidence of metastatic tumor formation in liver in mice carrying primary GC. Primary gastric cancer was induced in Helicobacter. felis (H. felis) infected INSp-GAS (expression of gastrin under insulin promoter) transgenic mice. A pooled primary gastric cancer cell (PGC) fraction was prepared from 10 mice that successfully generated tumor. These PGCs were transduced with a lentivirus carrying luciferase and GFP reporters under a ubiquitous cytomegalovirus promoter to allow visualization of the transduced cells in vivo, as well as distinguishing and separation of the transduced cells after transplanted into NOD/SCID mice. The PGCs were subcutaneously grafted into NOD/SCID mice to generate xenografted tumor, after which presence of CTCs in the circulation was assessed by flow cytometry for GFP, and formation of metastatic tumor in liver was examined by bioluminescence assay, 12 weeks after transplantation in mice with or without Curcumin treatment. Our results suggest that Curcumin may inhibit liver metastasis of GC through reducing CTCs.

## RESULTS

### Preparation of primary mouse gastric cancer cells

**Figure 1:**
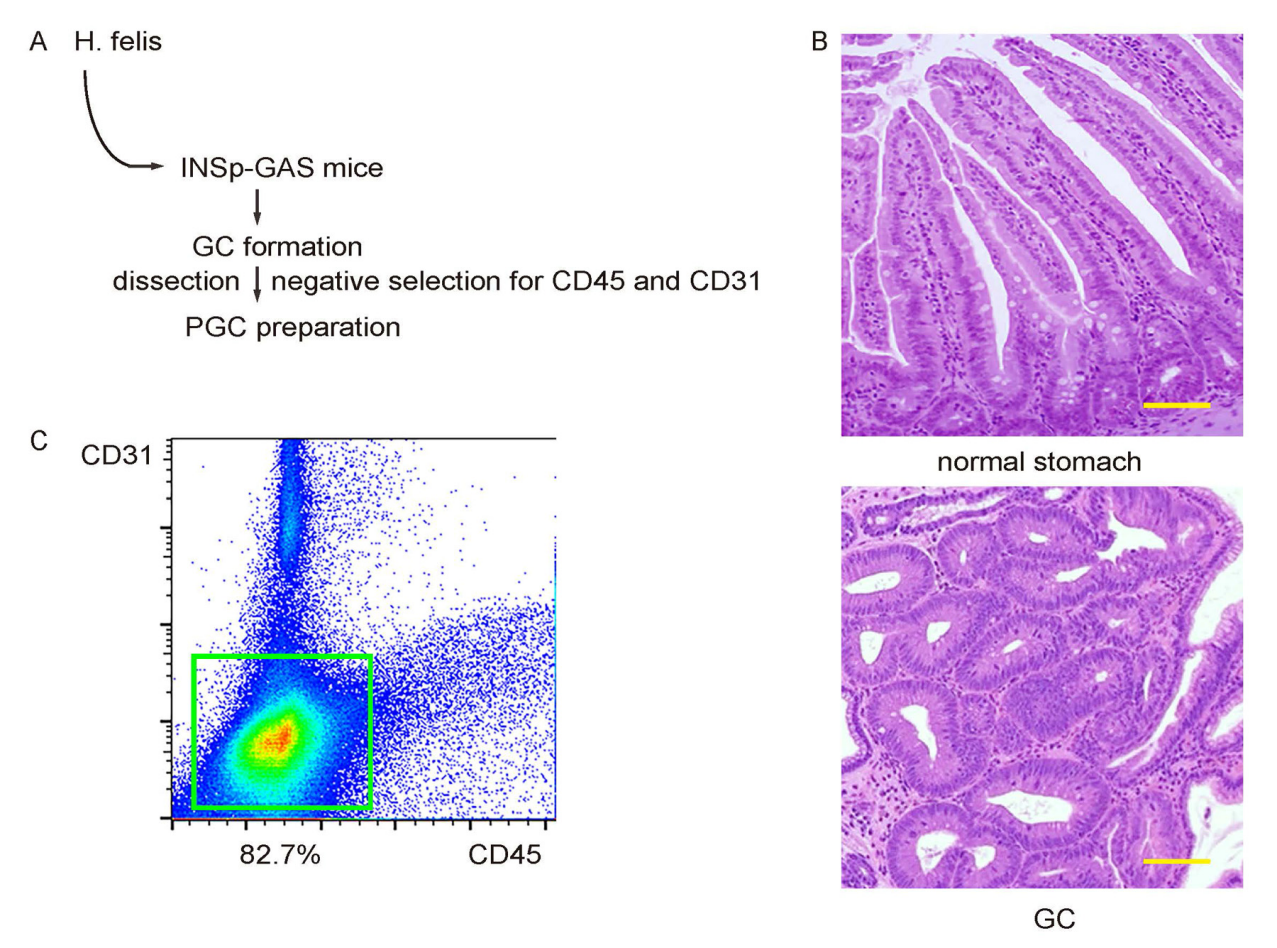
**Preparation of primary mouse gastric cancer cells.** (**A**) Schematic: H. felis was given to INSp-GAS (expression of gastrin under insulin promoter) transgenic mice, after which gastric tumor tissue was confirmed by histology after dissection out the formed tumor. (**B**) Representative histology of normal stomach and GC. (**C**) A pooled primary gastric cancer (PGC) cell fraction was prepared from 10 mice that successfully generated tumor, after a negative selection for CD45+ inflammatory cells, and CD31+ tumor endothelial cells. Scale bars are 100µm.

H. felis was given to INSp-GAS (expression of gastrin under insulin promoter) transgenic mice (Figure 1A), after which gastric tumor tissue was confirmed by histology after dissection out the formed tumor (Figure 1B). A pooled primary gastric cancer (PGC) cell fraction was prepared from 10 mice that successfully generated tumor, after a negative selection for CD45+ inflammatory cells, and CD31+ tumor endothelial cells (Figure 1C).

### Label PGC cells with GFP and luciferase

**Figure 2:**
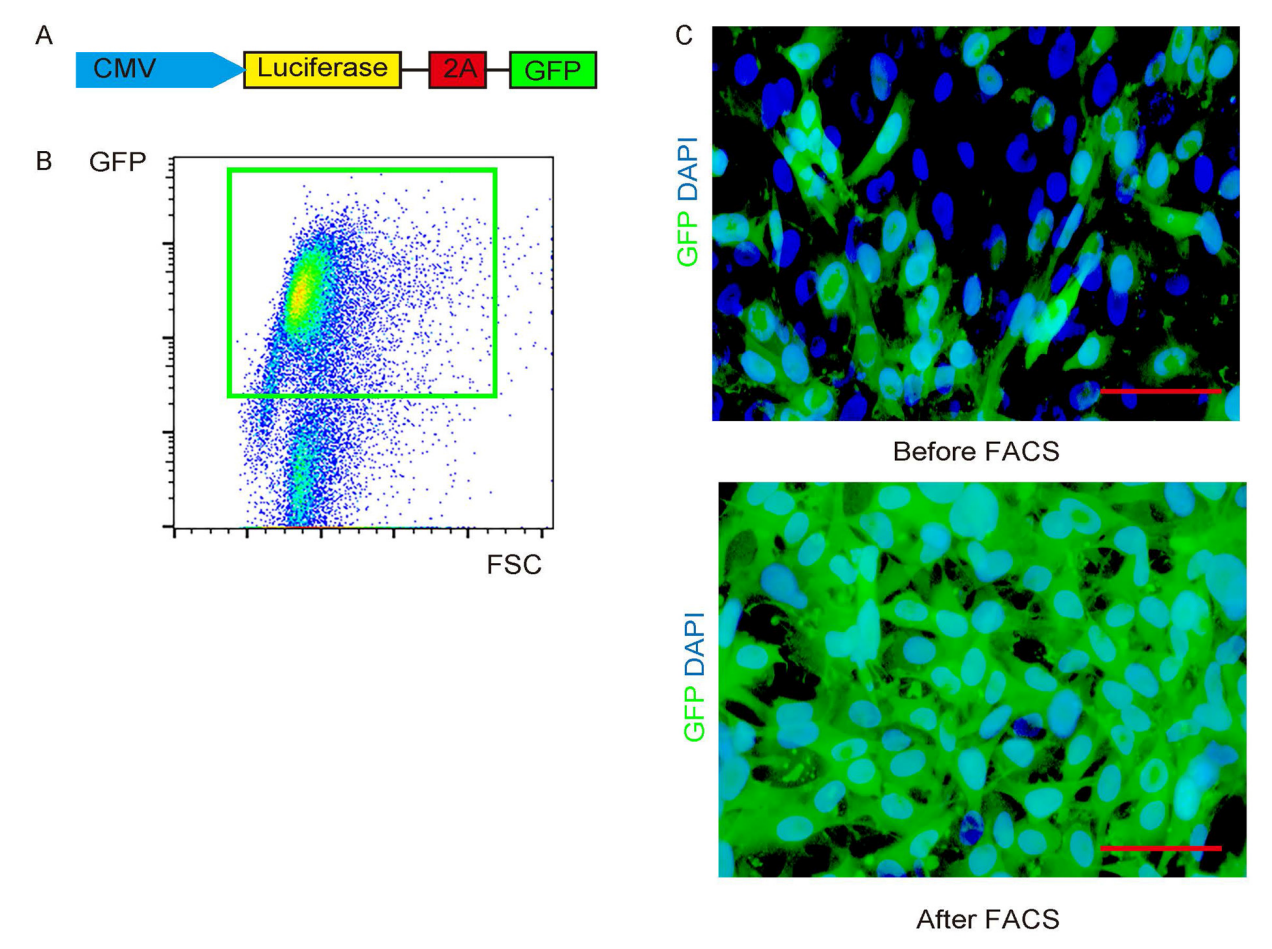
**Label PGC cells with GFP and luciferase.** (**A**) PGCs were transduced with a lentivirus carrying luciferase and GFP reporters under a ubiquitous cytomegalovirus promoter. (**B**) The transduced cells were visualized as green fluorescent cells, and thus can be purified by flow cytometry based on GFP. (**C**) Representative transduced cells in culture before/after FACS. Scale bars are 20µm.

PGCs were transduced with a lentivirus carrying luciferase and GFP reporters under a ubiquitous cytomegalovirus promoter to allow visualization of the transduced cells in vivo, as well as distinguishing and separation of the transduced cells after transplanted into NOD/SCID mice (Figure 2A). The transduced cells were visualized as green fluorescent cells. After purification by flow cytometry based on GFP (Figure 2B), the transduced cells were highly enriched in this fraction (Figure 2C).

### Curcumin reduces CTCs of s.c. grafted PGCs

**Figure 3:**
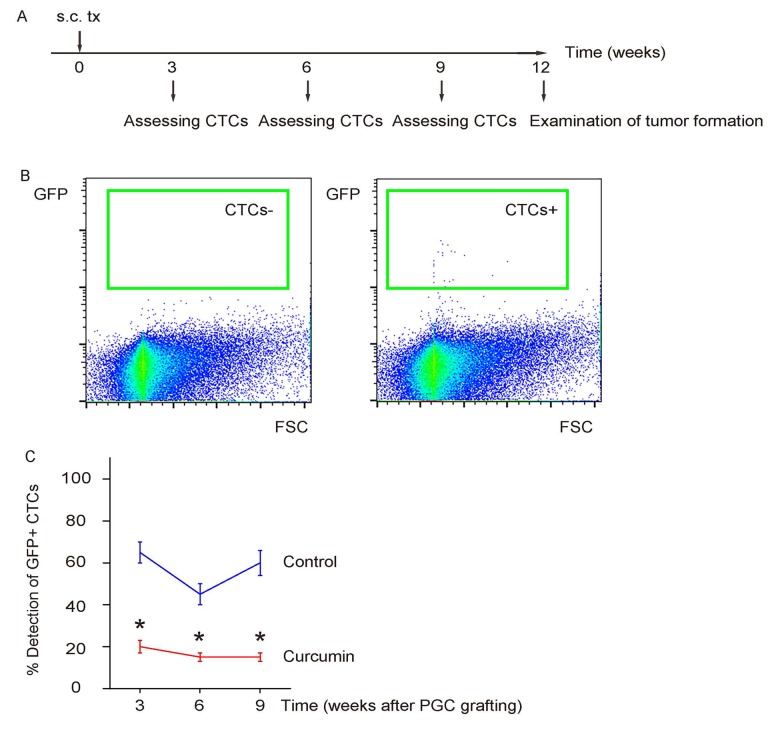
**Curcumin reduces CTCs of s.c. grafted PGCs.** (**A**) Schematic: The PGCs were subcutaneously grafted into NOD/SCID mice to generate xenografted tumor, after which some mice received liposomal Curcumin while some received liposome as controls. The presence of CTCs in the circulation was assessed by flow cytometry for GFP at 3, 6, 9 weeks after transplantation, 12 weeks after transplantation in mice with or without Curcumin treatment. (**B**) Representative flow charts for a negative detection or a positive detection of CTCs by flow cytometry. (**C**) Ratio of detection of CTCs. *p<0.05. N=30.

The PGCs were subcutaneously grafted into NOD/SCID mice to generate xenografted tumor, after which some mice received liposomal Curcumin while some received liposome as controls. The presence of CTCs in the circulation was assessed by flow cytometry for GFP at 3, 6, 9 weeks after transplantation, and formation of metastatic tumor in liver was examined by bioluminescence assay, 12 weeks after transplantation in mice with or without Curcumin treatment (Figure 3A). We found that Curcumin treatment significantly reduced the presence of CTCs (Figure 3B-C). Hence, Curcumin reduces CTCs of s.c. grafted PGCs.

### Curcumin reduces metastatic tumor formation in liver of s.c. grafted PGCs

**Figure 4:**
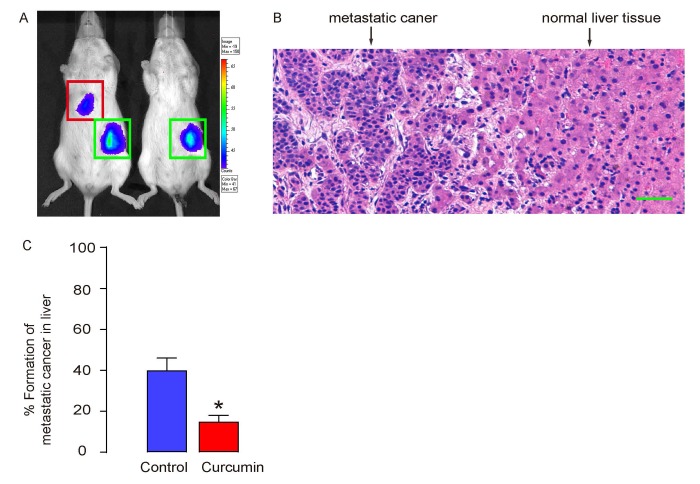
**Curcumin reduces metastatic tumor formation in liver of s.c. grafted PGCs.** (**A**-**C**) The formation of metastatic tumor in liver was assessed 12 weeks after transplantation in mice. (**A**) Representative images showing a positive and a negative case by bioluminescence in liver area. (**B**) A representative histological image showing metastatic cancer or normal liver. (**C**) Ratio of formation of metastatic tumor in liver. *p<0.05. N=30. Scale bar is 50µm.

The formation of metastatic tumor in liver was assessed 12 weeks after transplantation in mice. The positive case was first screened by bioluminescence in liver area (Figure 4A), and then confirmed by histology (Figure 4B). We found that Curcumin treatment significantly reduced the formation of metastatic tumor in liver (Figure 4C).

### Curcumin inhibits liver metastasis of GC through CXCR4

**Figure 5:**
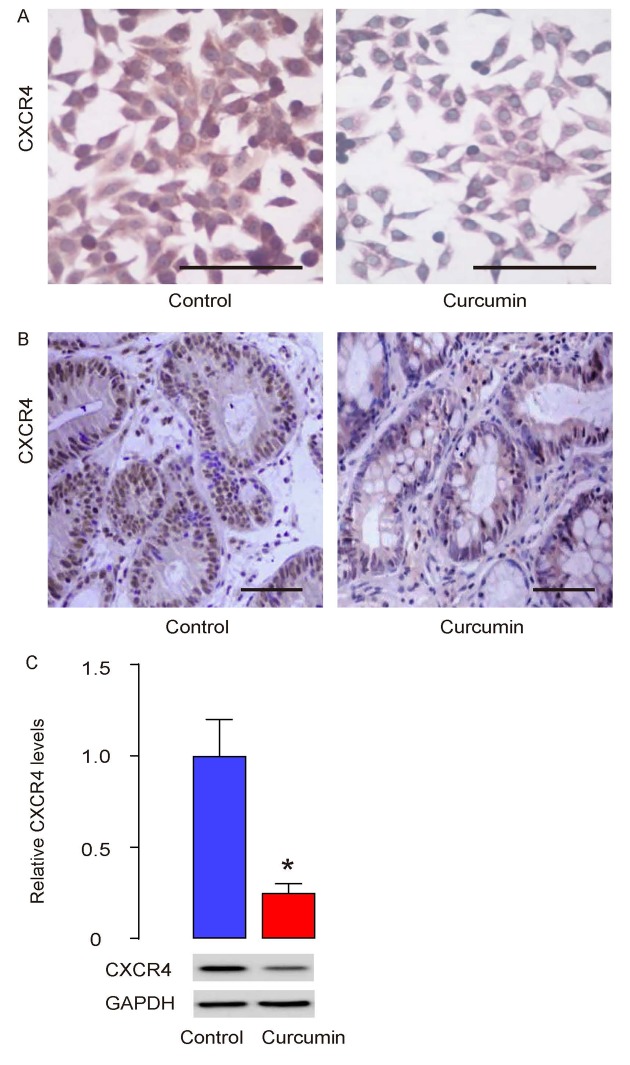
**Curcumin inhibits liver metastasis of GC through CXCR4.** (**A**) Cultured PGCs were treated with/without 0.5µmol/l Curcumin for 12 hours and then stained for CXCR4. (**B**) CXCR4 levels were also assessed in the formed tumor by s.c. PGC grafting in mice that had received Curcumin or control, by immunostaining (**B**), and by Western blotting (**C**). *p<0.05. N=30. Scale bars are 50µm.

Besides presence in circulation, CTCs are identified with a number of surface markers, among which CXCR4, the receptor for stromal cell -derived factor-1 (SDF-1), is the best characterized one in different types of cancer [5–7]. In our previous report, we have studied the CTCs in HCC and shown that CD90+CXCR4+ HCC cells may be CTCs and selective elimination of these cells may substantially improve the current HCC therapy by reducing cancer metastasis [8]. Thus, we analyzed whether Curcumin may affect CTCs by CXCR4. First, cultured PGCs were treated with/without 0.5µmol/l Curcumin for 12 hours and then stained for CXCR4. We found that Curcumin treatment reduced CXCR4 levels in cultured PGCs (Figure 5A). CXCR4 levels were also assessed in the formed tumor by s.c. PGC grafting in mice that had received Curcumin or control. We found that Curcumin treatment seemed to reduce CXCR4 levels in grafted tumor (Figure 5B). Finally, in dissected tumors from mice that had received Curcumin or control, the protein levels of CXCR4 were quantified by Western blotting, showing that the Curcumin treatment indeed reduced CXCR4 levels in grafted tumor (Figure 5C). Together, our data suggest that Curcumin may inhibit liver metastasis of GC through reducing CTCs through SDF-1/CXCR4 signaling.

## DISCUSSION

Among all types of GC, differentiated adenocarcinoma, poorly differentiated adenocarcinoma with a medullary growth pattern, and some special types like endocrine carcinoma and hepatoid carcinoma, are seemingly prone to metastasize to the liver. Recent evidence showed that CTCs are critical for the formation of metastatic tumor [27]. The CTCs need to disseminate from the primary tumor, in which an epithelial-mesenchymal transition process is required to occur to allow the tumor cells move to the periphery of blood vessels. Then the tumor cells need to penetrate and pass the endothelial cells-lined vessel wall, in which increases in vascular permeability and a series of modulation of adhesion molecules on the tumor cells, vessel endothelial cells and matrix cells should happen. SDF-1/CXCR4 signaling appears to play very important roles, since it regulates all steps in tumor progression, angiogenesis, metastasis, and survival [28].

In the current study, we first prepared the pooled PGC from a mouse model of GC. H. felis infection is the most important inducer to GC in human. Thus, the mouse GC model using H. felis is closest to the clinical manifestations of GC in human. However, H. felis alone does not typically induce GC in mouse, but it application to an INSp-GAS mouse model does induce aggressive GC [29]. Thus, this model was used here. PGCs from 10 mice were pooled together to reduce individual variation of the GC characteristics.

A negative selection for CD45 and CD31 was done to prepare PGCs, which significantly reduced the contamination of two major non-tumor cell types, inflammatory cells and endothelial cells in tumor. After this purification, the PGC fraction may only contain minor non-tumor populations, like mesenchymal cells, neurons, smooth muscle cells, etc. The effects of these minor populations to the interpretation of the data are very limited, compared to the important tumor-associated macrophages, lymphocytes, and tumor endothelia.

The curcumin was previously applied by both oral administration and intravenous injection. The latter was specifically in tumor treatment [30–32]. Also, it is noteworthy that intravenous injection of Curcumin has been used to empty gallbladder [33]. Here, we showed that intravenous Curcumin treatment significantly reduced the presence of CTCs and formation of liver tumor.

Mechanistically, Curcumin reduced CXCR4 expression in PGCs in vitro and in vivo, and thus likely inhibited metastasis of GC through suppression of SDF-1/CXCR4 signaling. The formation of metastatic cancer in liver may occur as early as 3 weeks after S.C. grafting, since CTCs were detected as early as that time point. Thus, Curcumin may be applied as early as possible to reduce the chance of formation of metastatic tumor. To the best of our knowledge, no previous studies have addressed such an anti-CTC role of Curcumin in GC, as well as its molecular basis.

Thus, our study suggests that Curcumin may inhibit liver metastasis of GC through reducing CTCs.

## MATERIALS AND METHODS

### Ethics

Experimental procedures in the current study have been approved by the research committee at Zhongshan Hospital of University of Fudan, and were carried out in accordance with the approved guidelines. Animal experiment protocols were approved by the Institutional Animal Care and Use Committee at Zhongshan Hospital of University of Fudan.

### Mice

NOD/SCID and INSp-GAS (expression of gastrin under insulin promoter) transgenic mice were both purchased from Jax mice (Bar Harbor, ME, USA). Female NOD/SCID mice of 10 weeks of age received subcutaneous grafting of 10^6^ tumor cells in the skin immediate on the top of the left limp. Male INSp-GAS mice of 10 weeks of age received Helicobacter. felis (H. Felis) as described [34] to form primary gastric cancer (PGC), which was confirmed by histology. Liposomal Curcumin (10mg/kg) or control liposome was injected to mice from the tail vein after tumor cell transplantation at a frequency of twice per week till the end of the experiment. The metastatic tumor formation in liver was determined with the IVIS imaging system (Xenogen Corp., Alameda, CA, USA) for bioluminescence assessment of the liver region, 12 weeks after transplantation, as has been described before [8].

### PGC cell preparation

The formed PGCs were dissected out and dissociated into single cells by incubation with Trypsin (0.25%) for 45 minutes at 37 °C. The single cell fraction of dissocated gastric tumor cells from 10 mice were then pooled together, incubated with APC-conjugated-anti-mouse CD45 (Becton-Dickinson Biosciences, San Jose, CA, USA) and Pacific blue-conjugated-anti-mouse CD31 (Becton-Dickinson Biosciences), and subjected to flow cytometry to isolate PGC fraction negative for both CD45 and CD31, to get rid of contaminated inflammatory cells and endothelial cells in the tumor.

### Labeling of PGCs with luciferase and GFP

The PGCs were transduced with a lentivector carrying luciferase and GFP reporters under a ubiquitous cytomegalovirus promoter to allow visualization of the transduced cells in vivo, as well as distinguishing and separation of the transduced cells after transplanted into NOD/SCID mice, as has been described before [8]. PGCs were cultured in Dulbecco’s modified Eagle’s medium (DMEM, Invitrogen, Carlsbad, CA, USA) supplemented with 10% fetal bovine serum (FBS; Sigma-Aldrich, St Louis, MO, USA) in a humidified chamber with 5% CO_2_ at 37 °C during the whole procedure of transduction, maintenance, and analysis.

### Detection of CTCs by flow cytometry

GFP-based detection of CTCs was performed by flow cytometry. GFP was determined by direct fluorescence. Flow cytometry was performed using a FACSAria (Becton-Dickinson Biosciences) flow cytometer. Negative controls were applied to remove background noise and to confirm positive cells. A total of 10^6^ RBC-depleted blood cells were analyzed for each time, and detection of more than 5 GFP+ cells was regarded as a positive case. Data were analyzed and presented using Flowjo software (Version 10, Flowjo LLC, Ashland, OR, USA).

### Histology and immunohistochemistry

H&E staining was performed as routine. CXCR4 immunostaining was carried out using a rabbit anti-CXCR4 antibody (Cell Signaling, Carlsbad, CA, USA). ABC method is used to develop the positive staining.

### Western blotting

The total protein was extracted from the tumor with RIPA buffer (Sigma-Aldrich) and quantified with BCA protein assay (Sigma-Aldrich). The rabbit anti-CXCR4 and rabbit anti-GAPDH (Cell Signaling) were applied for probing the target protein, then the membrane was incubated with the 2^nd^ antibody of HRP-labeled anti-rabbit (Dako, Carpinteria, CA, USA). The experiments were repeated for 5 times and the value was quantified with NIH ImageJ (Bethesda, USA).

### Statistical analysis

The data was statistically analyzed with GraphPad Prism 7 (GraphPad Software, Inc. La Jolla, CA, USA), as described [35–37]. The Student’s T test was performed to compare the data of 2 groups. The values were expressed as mean ± standard deviation (SD). When *p*<0.05, the data was considered as significant.
